# Yoga’s potential for promoting healthy eating and physical activity behaviors among young adults: a mixed-methods study

**DOI:** 10.1186/s12966-018-0674-4

**Published:** 2018-05-02

**Authors:** Allison W. Watts, Sarah A. Rydell, Marla E. Eisenberg, Melissa N. Laska, Dianne Neumark-Sztainer

**Affiliations:** 1 Division of Epidemiology and Community Health, University of Minnesota, Suite 300, 1300 S 2nd Ave, Minneapolis, MN 55454 USA; 2 Department of Pediatrics, University of Minnesota, 717 Delaware St. SE, Minneapolis, MN 55414 USA

**Keywords:** Yoga, Eating behaviors, Physical activity, Young adults, Mixed-methods

## Abstract

**Background:**

A regular yoga practice may have benefits for young adult health, however, there is limited evidence available to guide yoga interventions targeting weight-related health. The present study explored the relationship between participation in yoga, healthy eating behaviors and physical activity among young adults.

**Methods:**

The present mixed-methods study used data collected as part of wave 4 of Project EAT (Eating and Activity in Teens and Young Adults), a population-based cohort study in Minneapolis-St. Paul, Minnesota. Young adults (*n *= 1820) completed the Project EAT survey and a food frequency questionnaire, and a subset who reported practicing yoga additionally participated in semi-structured interviews (*n *= 46). Analyses of survey data were used to examine cross-sectional associations between the frequency of yoga practice, dietary behaviors (servings of fruits and vegetables (FV), sugar-sweetened beverages (SSBs) and snack foods and frequency of fast food consumption), and moderate-to-vigorous physical activity (MVPA). Thematic analysis of interview discussions further explored yoga’s perceived influence on eating and activity behaviors among interview participants.

**Results:**

Regular yoga practice was associated with more servings of FV, fewer servings of SSBs and snack foods, less frequent fast food consumption, and more hours of MVPA. Interviews revealed that yoga supported healthy eating through motivation to eat healthfully, greater mindfulness, management of emotional eating, more healthy food cravings, and the influence of the yoga community. Yoga supported physical activity through activity as part of yoga practice, motivation to do other forms of activity, increased capacity to be active, and by complementing an active lifestyle.

**Conclusions:**

Young adult yoga practitioners reported healthier eating behaviors and higher levels of physical activity than non-practitioners. Yoga should be investigated as an intervention for young adult health promotion and healthy weight management.

## Background

High rates of obesity, low levels of physical activity and poor dietary intake are highly prevalent around the world [1]. A critical time period for these problems is young adulthood, when overweight and obesity rates increase dramatically and behaviors such as physical inactivity and fast food consumption increase [2–5]. Few effective interventions exist for young adults [5, 6]; therefore, there is a need to identify and develop appropriate, innovative weight-related behavioral interventions that target this important life stage.

Yoga is an activity that combines physical movement, breathing, meditation, and mindfulness [7]. It is currently practiced by over 30 million US adults [8, 9], and has become increasingly popular in countries around the world [10, 11]. Initial studies suggest that yoga has a positive impact on health [12–15], including tentative evidence of benefits for weight-management [16, 17], and related factors such as disordered eating [18, 19], physical fitness [10], eating [20], sleep [8], mood [8], and stress [8]. The traditional yoga methods of inward focus, body awareness, and physical empowerment may promote a positive body image [21–23] and support health more holistically than traditional weight management interventions (e.g. exercise programs) [22]. Previous research has also shown that yoga is associated with engagement in physical activity for health rather than fitness or physical appearance [24], a motivation that may enhance yoga’s long-term maintenance. Prior research from our team suggests that frequency of yoga practice is associated with lower weight gain over time among young adults who practice yoga regularly, with greater effects observed for overweight young adults [17]. It is then of interest to explore the mechanisms by which yoga might promote healthy weight-related behaviors.

Yoga may have the added benefit of being more acceptable and adaptable than other forms of physical activity for those with large bodies or physical limitations [16]. However, yoga may also present unique challenges and barriers for larger individuals or those with body image concerns such as the use of mirrors in yoga facilities, which can exacerbate negative feelings and may be a barrier to participation [25]. The mainstream portrayal of yoga often includes slim women wearing tight fitting clothing that might limit participation by those who believe they do not fit that stereotypical image and lead to greater body dissatisfaction [26]. Since body image concerns [27], social stigma [28], physical activity preferences [29, 30], and perceptions/benefits of yoga may differ according to weight status, it is of interest to hear the perspectives of both overweight and non-overweight individuals. One previous qualitative study examined the weight loss experiences of 20 adults practicing yoga (mean age = 56.6 ± 9.6 years) and found that yoga was perceived to enable healthier eating habits and aid in weight loss [31]. Further research in a population-based sample of young adults that is diverse and includes those who are both overweight and non-overweight would be helpful to design and target interventions appropriate for young adults.

In response to the current lack of evidence to guide yoga interventions for weight-related health, this study aimed to explore the relationship between yoga and two behavioral mechanisms by which yoga may be helpful for weight management among young adults: healthy eating and physical activity. This study employed a mixed-methods design to: 1) examine associations between yoga practice, physical activity and eating behaviors in a large population-based sample of young adults, 2) explore potential mechanisms by which yoga is perceived to impact these behaviors through in-depth qualitative interviews with young adults who practice yoga, and 3) identify any similarities or differences in behaviors and perceived mechanisms of action across weight status.

## Methods

### Study design

This study utilized data collected from young adults as part of the fourth wave of Project EAT (Eating and Activity in Teens and Young Adults), a large, population-based cohort study on eating and weight-related health that first recruited 4746 adolescents in 1998–1999 from public middle and high schools in the Minneapolis-St. Paul metropolitan area of Minnesota, USA [32, 33]. Follow-up surveys were conducted online, by mail or by phone at five-year intervals (EAT-II, III, and IV). Additional details about the study design are described elsewhere [17, 34].

Survey data collected as part of EAT-IV were examined along with data collected from supplemental, semi-structured interviews conducted with a subset of survey participants. First, survey data were used to quantitatively examine associations between yoga participation and both physical activity and dietary intake behaviors. Second, data from semi-structured, qualitative interviews were used to complement survey findings and to allow for a phenomenological exploration of the mechanisms through which yoga practice might influence young adults’ diet and physical activity behaviors. Phenomenology is a method of inquiry that serves to uncover the meaning of individual experiences, predominantly through individual interviews [35]. All study protocols were approved by the University of Minnesota’s Institutional Review Board Human Subjects Committee and participants provided informed assent/consent.

### Young adult surveys

In 2015/16, 1820 young adults completed the EAT-IV survey including questions on yoga practice (out of 2770 invited to participate in this wave; response rate = 66%). The survey asked about a range of weight-related lifestyle behaviors and sociodemographic characteristics and included the addition of detailed questions about young adults’ yoga practice. The survey was tested in a sample of 103 young adults to examine test-retest reliability (test-retest correlations or percent agreements of survey measures are presented below). Details about the survey’s development are described elsewhere [17, 34]. Young adults also completed the previously validated 2007 Willett semi-quantitative food frequency questionnaire (FFQ) regarding their usual daily dietary intake over the past year [36, 37]. Nutrient intakes were determined by the Nutrition Questionnaire Service Center at the Harvard School of Public Health using a specially designed database, primarily based on the United States Department of Agriculture’s Nutrient Database for Standard Reference. The FFQ was completed by 1752 EAT-IV participants, however, data were not used if calculated energy intake was implausible (< 500 kcal/day or > 5000 kcal/day) or ≥ 20 items were incomplete (*n* = 150). A select number of dietary intake and physical activity variables that have relevance for young adult weight management were examined in the present study: fruits and vegetables, sugar-sweetened beverages, minimally nutritious snack foods, fast food, and moderate-to-vigorous physical activity [3, 38–40].

#### Measures

##### Yoga practice

Young adults who indicated they had ever done yoga (yes/no) and had done yoga in the past year (yes/no) were additionally asked, “On average, how frequently did you do yoga over the past year?” Response options were: < 30 min/week; 30 min to < 1 h/week; 1 to < 2 h/week; 2–3 h/week; 4–6 h/week; 7–9 h/week; 10+ hours/week. Respondents who indicated they had engaged in yoga at least 30 min per week in the past year were identified as *yoga practitioners* (test-retest agreement = 92%), all others were considered non-practitioners (i.e. did not practice regularly or had never done yoga). A continuous yoga variable was also created by converting each response option to the midpoint value (e.g., < 30 min/week = 0.25 h/week; 1–2 h/week = 1.5 h/week; 10 h/week = 11 h/week).

##### Fruit and vegetables (FV)

Daily servings of FV were derived from 43 different fruit and vegetable items included on the FFQ along with the option to write in items that were not listed. In most cases, a serving was defined as one-half cup of FV or one glass of juice. Responses were summed to obtain mean servings of FV per day.

##### Sugar-sweetened beverages (SSBs)

Daily servings of SSBs were derived from the FFQ and defined as the equivalent of one glass, bottle, or can of non-diet soda or fruit drink (i.e., not juice; including iced tea, lemonade, Kool-Aid®). Responses were summed to obtain mean servings of SSBs per day.

##### Minimally nutritious snack foods (snack foods)

Daily servings of snack foods were derived from the FFQ based on 24 minimally nutritious (i.e., high energy density, low nutrient density) food items commonly consumed as snacks, with the option to write in items that were not listed [41]. Snack foods included items such as chips, cakes, cookies, ice cream, sweets, pudding, crackers and baked goods. A serving of snack foods was defined by units such as 1 small bag, 1 pack, and 1 slice, as appropriate for the item. Responses were summed to obtain mean servings of snack foods per day.

##### Fast food frequency

A survey item asked participants, “In the past week, how often did you eat something from a fast food restaurant (like McDonald’s, Burger King, Hardee’s, etc.)?” Response options were never, 1–2, 3–4, 5–6, 7, and more than 7 times (test-retest = 0.54). Responses were collapsed into three categories (never, 1–2, and 3 or more times/week) based on increased health risk [39].

##### Moderate-to-vigorous physical activity (MVPA)

Survey items from the Godin-Shephard Leisure-Time Exercise Questionnaire were adapted to include both relevant exercise examples and six response options (0 to ≥6 h/week), and were validated against accelerometer-derived MVPA in a sub-sample of young adult participants in EAT-III [42, 43]. Hours spent in strenuous exercise (e.g., biking fast, aerobics, jogging, basketball, swimming laps, soccer, rollerblading) and moderate exercise (e.g., walking quickly, easy bicycling, skiing, dancing, skateboarding, snowboarding) were summed (test-retest *r* = 0.84) to obtain mean hours per week.

##### Weight status

Young adults self-reported their height and weight, from which body mass index (BMI, kg/m^2^) was calculated. Pregnant individuals were excluded due to normal changes in weight, dietary intake and activity behaviors. Self-report of height and weight (test-retest *r* = 0.95 for height and *r* = 0.98 weight) were previously validated in EAT-III in a subsample of 63 male and 62 female participants for whom height and weight measurements were completed by trained research staff [44]. Weight status was defined using current BMI guidelines for adults (overweight or obese: BMI ≥ 25 kg/m^2^) [45].

##### Covariates

Key sociodemographic variables that are of relevance to both yoga practice and the health behaviors of young adults included participants’ *age* (in years), *ethnicity/race* (White/Non-White), *gender* (male/female), and *educational attainment* (Bachelor’s degree or greater/Less than a Bachelor’s degree).

#### Analysis

The analytic sample for this study included 1820 young adults with complete data on their yoga participation. Sample characteristics are presented as means and percentages for continuous and categorical variables, respectively, with 95% confidence intervals. T-tests and Chi-square analyses were conducted to compare descriptive differences between yoga and non-yoga practitioners in the survey sample and among yoga practitioners between the survey and qualitative interview samples.

Linear and logistic regression models were run to determine associations between yoga practice and diet and physical activity variables. Models using a dichotomous yoga variable (regular yoga practitioners vs. non-practitioners) allowed for the comparison of behavioral differences between those who reported a regular yoga practice as compared to other young adults. Models using a continuous yoga variable were used to estimate associations comparing a 1-h difference in weekly yoga practice to a 1-unit difference in the dependent variables.

All models were examined unadjusted, and adjusted for age, gender, educational attainment and ethnicity/race. Models of FV, SSBs, and snack foods additionally adjust for total energy intake to help control for measurement error and individual differences in energy needs [46, 47]. Results of logistic regression models are presented as adjusted prevalence and standard errors when yoga was a dichotomous variable and as an odds ratio and 95% Confidence Interval (CI) when yoga was treated as a continuous variable. Results of linear regression models are presented as adjusted means and standard errors when yoga was a dichotomous variable and as unstandardized regression coefficients and standard errors when yoga was treated as a continuous variable. Adjusted prevalence/means were obtained with the margins command and group differences were tested using the Delta method with a *p*-value of less than 0.05 considered statistically significant. Interaction effects between regular yoga practice and weight status (overweight vs. non-overweight) were tested for each model. Interactions with a p-value< 0.10 were further examined in stratified models and results are presented as standardized regression coefficients and *p*-values.

Statistical adjustments were made to account for study attrition to allow for generalizations to the baseline school-based sample and to help adjust for possible selection bias. In all analyses, data were weighted using a response propensity method. Response propensities (i.e., the probability of responding to the EAT-IV survey) were estimated using a logistic regression of response at 15-year follow-up on predictor variables from the school-based survey. Study variables had a varying amount of missing data (0–13%) and all analyses were conducted with complete cases. The sample size for individual variables is shown in Table 1. All analyses were conducted with STATA statistical software, version 13.

**Table 1:** Demographic and lifestyle characteristics of young adult participants

	Survey Sample^a^			Interview Sample^b^	
	Non- Practitioners^c^ (*n* = 1523)	Yoga Practitioners^c^ (*n* = 297)		Yoga Practitioners^c^ (*n* = 46)	
	Mean/% (CI)	Mean/% (CI)	p-value^d^	Mean/% (CI)	p-value^e^
Demographic Characteristics^f^					
Age, yrs	31.1 (31.0–31.2)	30.8 (30.7–31.0)	.01	30.6 (20.1–31.1)	.16
Gender, % female	52.2 (49.7–54.8)	79.8 (74.8–84.0)	<.001	73.9 (59.2–84.7)	.28
Educational Attainment, % bachelor degree	49.3 (46.8–51.9)	69.9 (64.5–74.9)	<.001	76.1 (61.5–86.4)	.32
Ethnicity/race, % non-white	32.7 (30.3–35.1)	26.3 (21.6–31.6)	.03	17.4 (8.9–31.3)	.14
Weight status, % overweight (BMI ≥ 25 kg/m^2^)	62.2 (59.7–64.7)	44.4 (38.6–50.3)	<.001	51.1 (36.6–65.4)	.32
Lifestyle Characteristics^a^					
Fruit and Vegetables, servings/day [IQR = 2.8–6.8; *n* = 1592]	5.1 (4.9–5.3)	6.4 (5.9–6.8)	<.001	6.1 (5.1–7.1)	.30
Fast Food Frequency, %			<.001		
Never	37.5 (35.1–40.0)	55.2 (49.5–60.8)		52.2 (37.8–66.3)	.78
1–2 times/week	44.7 (42.2–47.2)	40.1 (34.6–45.8)		41.3 (27.9–56.1)	
3+ times/week	17.8 (16.0–19.8)	4.7 (2.8–7.8)		6.5 (2.1–18.6)	
Sugar-Sweetened Beverages, servings/day [IQR = 0–0.6; *n* = 1591]	0.6 (0.5–0.7)	0.3 (0.2–0.3)	<.001	0.1 (0.1–0.2)	.01
Snack Foods, servings/day [IQR = 0.6–1.6; n = 1592]	1.3 (1.2–1.3)	1.2 (1.1–1.3)	.10	1.0 (0.8–1.2)	.06
MVPA, hours/week [IQR = 1.3–6.6; n = 1820]	4.1 (3.9–4.3)	5.4 (4.9–5.8)	<.001	6.8 (5.8–7.9)	.003

BMI, Body Mass Index, MVPA, moderate-to-vigorous physical activity; CI, 95% confidence interval; IQR, Interquartile Range

^a^Survey sample includes the young adults who completed the Project EAT-IV survey, presented by yoga practitioner status

^b^Interview sample is the subsample of young adults who completed the Project EAT-IV survey who also participated in qualitative interviews

^c^Yoga practitioner defined as practicing yoga for ≥30 min/week over the past year; Non-practitioner is all others (less frequent yoga practice or no yoga practice)

^d^Difference between yoga practitioners and non-practitioners in the survey sample determined by ttest or chi^2^

^e^Difference between the interview sample and yoga practitioners in the survey sample determined by ttest or chi^2^

^f^Sample size for each variable varies slightly due to a small amount of missing data

### Qualitative interviews

#### Recruitment

Young adults were drawn from the survey sample based on their responses to survey questions on yoga practice, weight status and gender. Recruitment emails were sent to a convenience sample of survey participants who indicated that they practiced yoga on a regular basis (≥30 min/week in the past year). Recruitment emails described the study as related to young adults’ experiences practicing yoga and yoga’s perceived relationship to their behaviors and health (such as stress, eating habits, and body image). Recruitment was targeted to ensure that half the qualitative sample had a BMI ≥ 25 kg/m^2^. Recruitment was primarily conducted by email with some follow-up phone calls. More follow-up attempts were made for males in the overweight sample in an effort to obtain a more gender-balanced sample. A total of 46 young adults participated in interviews, 50% of whom were classified as overweight and 26% were male. Differences between those who participated in interviews and the full survey sample are described in the results and presented in Table 1.

#### Data collection

A semi-structured interview guide was first piloted with two yoga teachers and two young adults to make sure questions were clear, elicited in-depth discussion, and were acceptable to participants. Semi-structured questions inquired about young adults’ experiences, perceptions, and potential benefits of yoga practice. The present study explored responses to the interview questions about the impact of yoga on eating habits and physical activity. Participants were asked: 1) *Has yoga had an impact on your eating habits?* Prompts: *Think about the types of food, frequency of meals, portion sizes, state of mind while eating, and hunger cues. Can you explain how or why you think yoga impacted your eating habits? Was the change positive, negative or neutral (please explain)?;* and 2) *Has yoga had an impact on how active you are?* Prompts: *Have you become more or less active since beginning to practice yoga? Tell me more about that? Can you explain how or why you think yoga impacted your physical activity?*

Interviews were conducted by one of three female research staff members who were trained in qualitative research and interviewing techniques and had relevant experience practicing or teaching yoga. Interviews took place over a one-year period and ranged in length from 20 to 60 min. The majority of interviews took place in a private room on the University campus, while two interviews took place at community locations chosen by the participant and one interview occurred over Skype. Interviews were audio-recorded and written consent was obtained before commencing the interview. Following the interview, field notes were recorded. Young adults received a $50 Target gift card for participating.

#### Analysis

Transcripts of the audio-recorded interviews were coded and analyzed using directed thematic analysis [48, 49]. An initial coding scheme was developed based on the interview guide and the first transcript. The coding scheme was systematically and iteratively updated as additional transcripts were coded. The analysis allowed for new themes to emerge inductively so that young adults’ phenomenological descriptions of their experiences with yoga practice could be documented and interpreted [49]. Two to three research team members coded all transcripts independently. Each unique concept was coded and weekly team meetings were held to reach consensus on emerging themes and to organize/collapse themes where overlap was identified. Themes were also organized into positive, negative and neutral categories based on our interview guide questions, and patterns that emerged from the data that fit these categories. The coding scheme was maintained with a Microsoft Word file and coding was done by hand on hard copies of the interview transcripts, which were then used to compare coding among team members and to create a reconciled version. Coding from each reconciled interview transcript was entered into a Microsoft Excel file to tabulate the frequency of themes for each individual, the frequency of individuals endorsing each theme, and to compare differences in the frequency of individuals endorsing each theme by weight status and gender. Compelling quotes associated with each theme were also documented using Microsoft Word.

Results are presented to emphasize the unique ideas (coded as themes) that emerged from the interviews with young adults, with less emphasis on the number of young adults reporting each theme. However, to give the reader a sense of which themes may have been more salient and more commonly endorsed by this sample of young adults, the percentage of young adults who perceived yoga’s impact on their behaviors as positive, negative or neutral was reported and themes are presented in order of decreasing frequency (those themes that had the largest number of young adults endorsing them are presented first).

## Results

### Participant characteristics

The demographic and lifestyle characteristics of the survey sample by yoga participation status and the interview sample are presented in Table 1. In the survey sample, yoga practitioners were slightly younger (30.8 vs. 31.1 years of age, *p* = .01), and more were female (79.8 vs. 52.2%, *p* < .001), highly educated (69.9 vs. 49.3%, p < .001), white (32.7 vs. 26.3%, *p* = .03) and non-overweight (62.2 vs. 44.4%, p < .001) than non-practitioners. The characteristics of yoga practitioners who participated in qualitative interviews were compared to yoga practitioners in the larger survey sample from which they were drawn to determine the representativeness of the interview sample. Interview participants reported more hours of MVPA per week (6.8 vs. 5.4 h, *p* = 0.003) and lower consumption of SSB (0.1 vs. 0.3 servings, p = .01) than yoga practitioners in the survey sample. No other statistically significant differences were found between interview participants and non-participants, however, due to the small sample of interview participants, comparisons will have limited power. All descriptive, sociodemographic differences between the two groups are shown in Table 1.

### Associations between regular yoga practice and dietary and physical activity behaviors

Regular yoga practice was positively associated with healthier dietary intake and greater physical activity in unadjusted models and models adjusted for demographic characteristics. Results from unadjusted models were similar to adjusted models in effect size and statistical significance (data not shown). Results from regression models adjusted for demographic characteristics (Table 2) showed that, as compared to non-practitioners, yoga practitioners reported consuming, on average, one more serving of FV (6.2 vs. 5.2 servings/day), 0.3 fewer servings of SSBs (0.4 vs. 0.7 servings/day) and 0.2 fewer servings of snack foods (1.1 vs. 1.3 servings/day), were less likely to eat from a fast food restaurant (8% vs. 21% reported consuming fast food 3+ times/week), and participated in two more hours of MVPA (5.9 vs. 4.0 h/week).

**Table 2:** Predicted prevalence or mean of dietary and physical activity behaviors comparing yoga practitioners and non-practitioners

	Non-Practitioners	Yoga Practitioners			
	Mean/% (SE)	Mean/% (SE)	**Δ**	[95% CI]	p-value
Fruit and Vegetables, mean servings/day	5.2 (0.1)	6.2 (0.2)	1.0	[0.5, 1.5]	<.001
Fast Food Frequency, %					
Never	32.8 (1.3)^a^	47.2 (3.7)^b^	14.4	[6.9, 22.0]	<.001
1–2 times/week	46.8 (1.5)^b^	42.2 (3.7)^b^	−4.6	[−12.2, 3.0]	
3+ times/week	20.4 (1.3)^a^	7.6 (2.2)^b^	−12.9	[−17.7, −8.0]	
Sugar-Sweetened Beverages, mean servings/day	0.7 (0.03)	0.4 (0.1)	−0.2	[−0.4, − 0.1]	<.001
Snack Foods, mean servings/day	1.3 (0.03)	1.1 (0.1)	−0.2	[−0.3, − 0.1]	.01
Moderate-to-Vigorous Physical Activity, h/week	4.0 (0.1)	5.9 (0.3)	1.9	[1.3, 2.5]	<.001

SE, standard error; CI, confidence interval

Models control for age, gender, race/ethnicity and education, and are weighted for non-response. Models for fruits and vegetables, sugar-sweetened beverages, and snack foods are additionally adjusted for energy intake

^ab^Different letter superscripts represent a statistically significant difference between yoga practitioners and non-practitioners for each fast food frequency level, *p* < .05. For example, a significantly higher proportion of yoga practitioners report *never* consuming fast food than do non-practitioners, while there is not significant difference between yoga practitioners and non-practitioners in reporting of fast food consumption 1–2 times per week

When frequency of yoga practice was treated as a continuous variable in models adjusted for demographic characteristics, similar results were found. Specifically, for each additional hour of reported yoga practiced per week, MVPA was higher by 0.9 h per week (b = 0.87, SE = 0.10, *p* < .001), fruit and vegetable intake was higher by 0.6 servings per day (b = 0.60, SE = 0.10, p < .001), consumption of sugar sweetened beverage (b = − 0.10, SE = 0.03, p < .001) and snack foods (b = − 0.08, SE = 0.03, *p* = .002) were both lower by 0.1 servings per day, and the odds were lower for consuming fast food intake 1–2 times/week (OR = 0.83, 95% CI 0.73–0.94, *p* = .005) and 3+ times/week (OR = 0.44, 95% CI 0.30–0.65, *p* < .001) as compared to never consuming fast food.

### Differences in associations by weight status

Interactions by weight status were found for MVPA (*p* = .07) and snack food consumption (p = .002). In stratified models, those practicing yoga also reported greater weekly MVPA in both weight status groups, however, the difference in hours of weekly MVPA between yoga practitioners and non-practitioners was greater for overweight young adults (Δ = 2.5 h [95% CI = 1.6, 3.4], *p* < .001) than for non-overweight young adults (Δ = 1.31 h [95% CI = 0.4, 2.2], *p* = .01). Furthermore, yoga practice was negatively associated with snack food consumption for overweight young adults only; yoga practitioners who were overweight reported consuming approximately half a serving fewer snack foods than non-practitioners who were overweight (Δ = − 0.4 servings/day [95%CI = − 0.6, − 0.2], p < .001). The remaining interactions were non-significant, indicating that associations did not differ for overweight and non-overweight practitioners (data not shown).

### Yoga’s perceived impact on dietary and physical activity behaviors

To complement survey data, young adults were asked in qualitative interviews about their perceptions of if and how yoga influenced their eating behaviors and physical activity level. Most young adults interviewed (90%) indicated that yoga had a positive impact on their eating behaviors. The remaining young adults were unsure or thought yoga had no impact on their eating. Three quarters of young adults interviewed (75%) indicated that yoga had a positive impact on how active they were. The remaining young adults described a neutral impact. No one reported a negative impact of yoga on his or her eating behaviors or physical activity. Themes that emerged illustrating how young adults viewed yoga as influencing their eating behaviors and physical activity level are described and expanded upon below and in Table 3.

**Table 3:** Themes identified by young adults: The influence of yoga practice on eating and physical activity (n = 46)

Theme		Example Quotes
Eating Behaviors	Motivated to make healthier choices	“Because I know I’m trying to improve myself by doing yoga and I think I shouldn’t go and get a Subway sub. Or if I do, I get the healthier version. [Yoga] puts me in a mindset, like, I’m trying to be better, so then I eat better”
	More mindful eating	“[As a result of yoga] I tend to choose the things that fuel [my body] rather than the things that are just available and easy to eat and taste delicious.”
	Management of stress and emotional eating	“When you’re feeling less anxiety, more peaceful, more hopeful about things, then, you don’t see the need to consume everything in your house.”
	More healthy food cravings	“After you’re sweating for 60 min [in yoga], I get out and crave good food.”
	The yoga community	“I became closer friends with people that did yoga who also happened to eat healthier. I spent more time in places where people were interested in being healthy.”
Physical Activity	Activity due to yoga practice	“In the winter I don’t do any sports, so [Yoga’s] my one way of moving.”
	Motivated to do other forms of physical activity	“[Yoga] gave me enough confidence to go out and join the gym and get a personal trainer, and I don’t think I would have necessarily done those steps had it not been for starting out with the yoga and seeing results with that and feeling more comfortable with myself.”
	Improved capacity to be physically active	“I coach basketball…before that, I couldn’t get out and do stuff, and I started yoga, and I’m able to… run up and down with the high school kids now and play basketball with them. So [yoga] helps me be more active.”
	Complemented an already active lifestyle	“[Yoga] definitely makes my body feel better… but I definitely try to be very active, so in my mind I would say my activity level encompasses yoga versus yoga making me more active.”

#### i. Eating behaviors

Five themes emerged related to yoga’s impact on eating behaviors: 1) Motivation to make healthier choices; 2) More mindful eating; 3) Management of stress and emotional eating; 4) More healthy food cravings; and 5) The yoga community**.** No differences in themes were noted by gender and only a small difference was found by weight status (described below). Supporting quotes for each theme are presented in Table 3.

##### Motivation to make healthier choices

Greater motivation for healthy eating was primarily related to the types of foods participants selected to eat. This motivation was commonly discussed by young adults, particularly related to selecting healthier foods and beverages or avoiding unhealthy foods and beverages when practicing yoga because they did not want to negate the benefits of the workout – i.e. yoga was a healthy choice they had made and they wanted to continue with a healthy choice when deciding what to eat following class. In addition to the types of foods that young adults reported eating, participants also talked about being motivated to cook healthy meals after a yoga class or selected lighter foods or smaller portion sizes before and after class to avoid feeling uncomfortable.

##### More mindful eating

Greater mindfulness was a similar but distinct concept from greater motivation to make healthy choices that was related more to nourishment and their state of mind rather than concepts of ‘healthy/unhealthy’ or ‘calories’. It was also related to being psychologically present while eating and having greater awareness and appreciation for food. Young adults perceived yoga to promote increased awareness of their bodies’ needs, which helped them to select foods that nourished their bodies and provided fuel, be more attentive to signals of hunger or fullness, and have greater presence, appreciation and focus on their food while eating as a consequence of practicing yoga. For example, one young adult said: *“[Yoga] does help to notice and be more in tune with your body and what it needs and what it wants…You tend to pay more attention to when you’re actually hungry, and you eat maybe a little bit less.”*

##### Management of stress and emotional eating

Young adults also described fewer instances of stress-related or emotional eating as a result of their yoga practice. Young adults described a change to their eating because of improvements in their mood following yoga practice and felt that yoga improved their stress levels and thus prompted better habits*: “When I’m stressed myself, I usually overeat, I tend to get fast food…with the stress reduced [from yoga], it helps me calm down and take the time to make meals and cook at home.”*

##### More healthy food cravings

Some young adults described that yoga led their bodies to crave healthier foods after class. This effect was described as a physiological response rather than an intentional decision to simply make healthier food choices.

##### The yoga community

The yoga community was described as one that exposed participants to a different social circle, where healthy eating was commonplace. The yoga community was also described as one that encouraged feelings of connection, which translated into a better sense of one’s connection to food and the community: *“[Yoga] is about connecting the mind and the body, which in turn, connects the body to food and the food to the community”.*

##### Comparison across weight status

The idea of making healthier choices specifically because someone does not want to *“undo the workout”* (i.e., consume more calories than they expended in a workout) was expressed by twice as many young adults categorized as overweight (*n* = 18) than non-overweight (*n* = 9). For example, as one young adult categorized as overweight noted: *“[After yoga] I’d go home and then make a small meal, because I don’t want to undo the work … [healthy food] doesn’t make you feel like you’ve wasted all that effort.”* No other differences by weight status in the frequency of young adults endorsing a theme related to eating behaviors were apparent.

#### ii. Physical activity

Four themes emerged related to yoga’s impact on physical activity: 1) Activity due to yoga practice; 2) Motivation to do other forms of physical activity; 3) Improved capacity to be active; and 4) Complemented an already active lifestyle. No differences were apparent by gender or weight status. Supporting quotes for each theme are presented in Table 3.

##### Activity due to yoga

Many young adults described being more active because they were active in their yoga practice. For some, yoga was their only form of physical activity, while for others, it was an additional form of physical activity that contributed to an increase in their overall activity level.

##### Motivation to do other forms of physical activity

Young adults also said that yoga motivated them to be more active. This increased motivation was attributed to yoga’s contribution to their enjoyment of other activities, their energy to be active, and their interest in trying other activities. For example, yoga provided some young adults with the skills and/or experience to be confident in taking part in other forms of physical activity.

##### Improved capacity to be active

An increase in physical strength and flexibility from yoga was perceived by many young adults to result in fewer injuries and greater mobility so that they were less likely to be sidelined from other forms of physical activity. Greater mobility and strength as a result of yoga also allowed some young adults to try other physical activities for the first time.

##### Complemented an already active lifestyle

Some young adults said that they were already very active and that yoga didn’t add any additional activity to their week. For example, if they weren’t practicing yoga, their time would be spent in some other form of activity. However, these individuals felt that yoga complemented their other activity. For example, some young adults described the stretches and movement of yoga as a good counter balance to the mental and physical impact of running on their bodies.

## Discussion

Results from this mixed-methods study reveal a positive association between yoga, healthy eating and physical activity. Using population-based survey results, findings indicate that young adults who practiced yoga had higher quality diets and greater moderate-to-vigorous physical activity levels than non-practitioners. These findings were further corroborated in qualitative interviews where young adults who practiced yoga described a positive impact on their dietary intake through increased motivation to eat healthy foods after class, being more mindful of what their bodies needed, better management of emotional eating, craving healthier foods, and the social influence of their yoga community. They also described a positive impact on their physical activity level because of a greater interest and ability to participate in physical activity (including activities beyond yoga). These benefits were seen among young adults across weight status, with some evidence for a particular benefit to those of higher weight status (e.g., yoga was associated with lower snack food consumption only among young adults categorized as overweight).

Among many people, being active and eating healthfully are commonly co-existing behaviors [50, 51]. However, yoga deserves attention as an activity to also promote healthy eating because of its emphasis on greater body awareness and mindfulness, which may translate to more mindful eating and better stress management (e.g. reduce overeating); a relationship supported by the perceptions of young adults in this study and findings from previous research [31, 52, 53]. Although participants were specifically prompted to reflect on mindful eating,, it was not clear if participants would report a positive relationship between yoga and mindful eating or how they would describe their experiences with yoga and mindful eating, if any. Our results also support findings from a previous qualitative study of adults who lost weight or were trying to lose weight while practicing yoga, who reported an increase in mindful eating, changes in food choice, decreased emotional and/or stress eating, and an influence from the culture of the yoga community [31]. Our findings further expand on this work by demonstrating similar benefits perceived by young adults and those not actively trying to lose weight. These positive benefits of yoga on health behaviors could have implications for interventions where both physical activity and eating behaviors need to be targeted. However, there remain questions about how young adults who practice yoga might differ from the general population in ways that independently influence their eating and activity behaviors. Longitudinal and intervention studies are needed to quantify the effects of yoga on diet quality and other measures of eating behavior to determine if eating behaviors can change as a result of yoga, if changes are sustained over time, and if changes lead to improved weight outcomes.

Although different forms of yoga can range from requiring very little movement to being quite vigorous (with varying impact on energy expenditure), evidence from this study suggests that young adults who practice yoga are achieving more vigorous activity levels, perhaps because they are practicing a vigorous form of yoga or, as our qualitative findings suggest, because yoga can be a “gateway” to more activity (e.g. yoga might improve mobility and fitness or increase motivation to try other forms of activity). Yoga as a form of entry to more vigorous activity could be particularly beneficial for the large number of young adults who are inactive or do not achieve recommended levels of physical activity [54, 55]. These findings warrant further investigation and suggest yoga may be a unique physical activity intervention to consider for young adults who are inactive or struggle with their body weight and are interested in being more active. Stronger associations with MVPA and some eating behaviors (e.g. snack food consumption) among overweight yoga practitioners in the present study may also explain our earlier findings that show stronger effects of yoga on BMI change among overweight as compared to non-overweight young adults who practice yoga [17].

Strengths of this study include the integrated use of both quantitative and qualitative data to better understand how yoga practice may benefit young adults’ diet and physical activity behaviors among both overweight and non-overweight individuals, and the use of a large, population-based sample. Limitations of this study include the cross-sectional and self-reported nature of the survey data. These limitations make it difficult to determine cause and effect and may lead to some degree of bias. For example, a third factor, such as an interest in overall health, may be independently related to participating in yoga and making healthier eating decisions, explaining the observed positive associations between yoga and dietary quality. In addition, those doing yoga could be more physically active from practicing yoga or from engaging in other types of MVPA and we could not distinguish between these two scenarios in our study. Triangulating survey results with in-depth, qualitative data mitigates some of these limitations. Finally, the qualitative interviews were conducted with young adults living in the Minneapolis St-Paul metropolitan area and may not reflect the views of other young adults in the United States, particularly in rural areas. Similarly, interpretations of the data and the conclusions drawn are influenced by the characteristics, experiences, perspectives, and expertise of the research team.

## Conclusions

Results from this study suggest that yoga shows promise as an intervention to improve the weight-related health of young adults through healthier eating patterns and physical activity. These findings are preliminary and suggest cross-sectional associations are present, however, further research is needed to determine if yoga is causally related to healthy eating and physical activity behaviors. Future studies are needed to explore other possible mechanisms by which yoga can improve weight-related health such as through improved body satisfaction, sleep, stress, and mood [56–59]. The intention of yoga to empower and support a positive sense of self makes it a particularly relevant strategy for the healthy weight management of women and those who are at increased risk for poor body image, however, caution is needed to avoid potential harms to individuals previously identified in the literature, namely, the use of unhealthy weight control behaviors among young adults [60], and women with obesity [11]. Additional attention is also needed to identify the dose of yoga that will produce benefits [61], and to the structural aspects of yoga that may currently cause discomfort among people of various body sizes, particularly those who have not practiced yoga previously. Examples of these structural barriers include mirrored studios or a lack of private spaces for changing that can exacerbate negative feelings and be a barrier to participation [25]. Future research that employs longitudinal and experimental designs in diverse populations will be needed to determine the efficacy of yoga interventions for behavioral and health outcomes.

## Abbreviations

BMI: Body Mass Index
FFQ: Food Frequency Questionnaire
FV: Fruit and Vegetable
MVPA: Moderate to Vigorous Physical Activity
SSBs: Sugar Sweetened Beverages
